# Virtual Orientation Tools (VOTj): Fiji plugins for object centering and alignment

**DOI:** 10.17912/micropub.biology.001221

**Published:** 2024-06-08

**Authors:** Sankeert Satheesan, Jochen Gehrig, Laurent S.V. Thomas

**Affiliations:** 1 Acquifer, Luxendo GmbH, Heidelberg, Germany; 2 Centre for Organismal Studies (COS), Heidelberg University, Heidelberg, Germany

## Abstract

Standardizing image datasets is essential for facilitating overall visual comparisons and enhancing compatibility with image-processing workflows. One way to achieve homogeneity for images containing a single object is to align the object to a common orientation. Here, we propose the Virtual Orientation Tools (VOTj): a set of Fiji plugins to center and align an object of interest in images to a vertical or horizontal orientation. To process an image, the plugin requires either a mask outlining the object or a rough annotation of the object directly drawn by the user in the image. The current object orientation is retrieved using Principal Component Analysis (PCA), from which the optimal alignment is derived. The plugins support multi-dimensional images to allow, e.g., aligning individual time points of a time-lapse. The tools can be used for a variety of samples and imaging modalities. Besides, the plugins enable the interactive alignment of a list of images from a directory for batch execution and can be included in custom image-processing workflows using macro-recording.

**Figure 1. Virtual Orientation Tools (VOTj) for object alignment.**
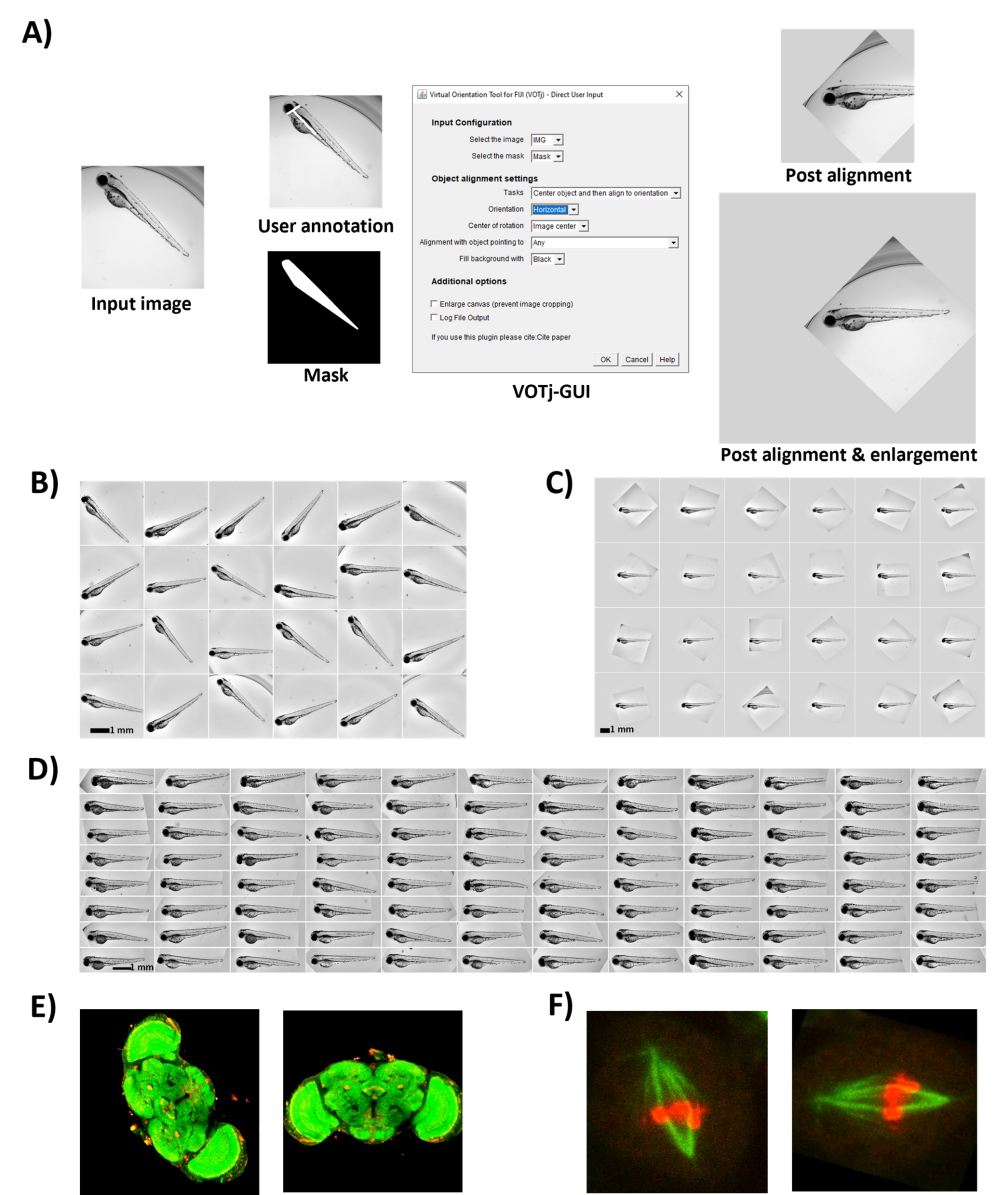
(A) Left: Overview of the VOTj plugin workflow and its application in aligning and centering a zebrafish embryo with the corresponding mask or user annotation. Center: User interface panel. Right: Resulting output images with and without the option of enlarging the canvas to prevent cropping original image regions. (B) An illustrative subset of zebrafish embryos imaged in a 96-well plate. Without dedicated mounting, these embryos adopt random orientations. (C) Corresponding images after transformation by the Virtual Orientation Tool plugin, using the option to enlarge the canvas. (D) Montage showing the centered and aligned zebrafish embryos across a full 96-well plate. The aligned images were cropped to display only the embryos. (E-F) The tool can be used to align various samples and is compatible with multi-dimensional images such as Z-stacks and time-lapse images, as shown in (E, F) for the alignment of a fluorescently labeled fly brain and a cell in mitosis, respectively. Input stack (left) and aligned stack (right). All images are background-corrected.

## Description

Object alignment and centering often constitute key steps in an image processing workflow, ensuring a uniform dataset for subsequent processing and visualization. Working with aligned images enhances the results of object detection and classification and enables straightforward visual comparisons (Chen et al., 2022; Erickson et al., 2007; Han et al., 2022) . Manually aligning images is time-consuming and prone to variability. To address these challenges and to provide a user-oriented solution in a familiar software environment, we developed a set of plugins for ImageJ/Fiji (Schindelin et al., 2012) : the Virtual Orientation Tools (VOTj).

The developed plugins allow both centering and alignment of an object of interest in an image, providing a mask or a rough annotation outlining the object of interest ( Fig. 1A ). Based on the annotation, the plugin computes the translation and/or rotation to position the object of interest in the center of the image and to align its main elongation axis with the vertical or horizontal axis, as decided by the user. The original orientation of the object of interest is computed using Principal Component Analysis (PCA). We used here the implementation from OpenCV, available in Fiji via the IJ-OpenCV update site (Domínguez et al., 2017; Hafiz Zia Ur Rehman et al., 2018; *OpenCV: Introduction to Principal Component Analysis (PCA)* , n.d.)

The plugins feature a user-friendly interface guiding the users through various configurations, starting with selecting the input image and its corresponding mask. Users can either provide an existing binary mask outlining the object or annotate the object of interest in the input image using the brush tool ( Fig. 1A ). For alignment, the plugin offers three options: centering, alignment (horizontal or vertical), or a combination of both. Rotational and translational transformations during alignment cause the original image to exit the original canvas. The plugin supports the option to enlarge the output image to address this issue ( Fig. 1A ). Additionally, most elongated objects present a natural directionality (head/tail, top/bottom). To ensure consistent alignment across multiple images, the tool supports, in addition to the axis of alignment (horizontal/vertical) the option to define in which direction the object should be aligned along that axis ( Fig. 1D ), ( Fig. 1A ). If not specified, the plugin aligns the object with the closest axis regardless of direction.

VOTj can be a preprocessing step for a diverse range of image analysis workflows, e.g., for time-lapse imaging, to align the object of interest to a common orientation at each time point. Here, we illustrated the application of VOTj for the rotational alignment of zebrafish embryos in a 96-well plate ( Fig. 1B ). Although several mounting systems have been reported for such specimens, they require additional sample preparation and equipment (Gehrig et al., 2018) . Here, the sample preparation is minimal and allows straightforward imaging. The random orientation of the specimen is subsequently corrected using VOTj to ensure a uniform position and rotational alignment of the embryos ( Fig. 1C ), thereby facilitating overall visual comparison ( Fig. 1D ).

The plugin has two modes of operation, single and batch, allowing users to align individual images or entire folders of images. The plugin is specifically adapted to align a single object in each image, and the alignment propagates to multiple dimensions. For timelapse, each timepoint can be aligned separately, while channels and Z slices are typically aligned following the same transformation (Fig.1E & 1F). The plugin supports batch processing and is macro recordable, ensuring seamless integration into existing workflows and broadening its applicability.

## Methods

The Virtual Orientation Tool can be installed in Fiji by activating the “Virtual-Orientation-Tools-VOTj” and “IJ-OpenCV” update sites.

Documentation and source code are available in Extended Data and on GitHub ( https://github.com/sankeert1999/Virtual-Orientation-Tools-VOTj ).

A video tutorial is available in Extended Data and on YouTube ( https://youtu.be/WHeDhn1Mnpc ).

The zebrafish embryos for Figure 1 were imaged in a 96-well plate using an Acquifer IM (Bruker, Heidelberg), an automated fluorescence widefield screening microscope equipped with a 4x objective (Nikon). The images are available on Zenodo ( https://doi.org/10.5281/zenodo.11093963 ).

A preconfigured Fiji bundle for Mac and Windows is also archived on Zenodo ( https://doi.org/10.5281/zenodo.11093038 ).

The FlyBrain ( http://imagej.net/images/flybrain.zip ) image in Fig. 1E. and the Mitosis ( http://imagej.net/images/Spindly-GFP.zip ) image in Fig. 1F. are both ImageJ sample images (credit NIH).

## Extended Data

Description: A video tutorial showcasing how to utilize the plugin. (Also posted on YouTube). Resource Type: Audiovisual. DOI: 10.22002/4g5rz-syt88

Description: Copy of the GitHub repository. Resource Type: Software. DOI: 10.22002/sspxt-tst47
